# Artificial Intelligence in Population-Level Gastroenterology and Hepatology: A Comprehensive Review of Public Health Applications and Quantitative Impact

**DOI:** 10.1007/s10620-025-09452-7

**Published:** 2025-10-24

**Authors:** Hareesha Rishab Bharadwaj, Dushyant Singh Dahiya, Priyal Dalal, Muhtasim Fuad, Hafiz Ali Raza, Muhammad Ibrahim, Arkadeep Dhali, Fariha Hasan, Balamrit Singh Sokhal, Karan Yagnik, Bhanu Siva Mohan Pinnam, Farhan Gohar, Hassam Ali

**Affiliations:** 1 Royal Stoke University Hospital, University Hospitals of North Midlands NHS Trust, Stoke-on-Trent, ST4 6QG UK; 2 Division of Gastroenterology, Hepatology & Motility, University of Kansas School of Medicine, Kansas City, KS 67214 USA; 3 School of Medicine, The University of Central Lancashire, Preston, PR1 2HE UK; 4 Faisalabad Medical University, Faisalabad, 38000 Pakistan; 5 Bannu Medical College, Bannu, 02816 Pakistan; 6 Academic Unit of Gastroenterology, Royal Hallamshire Hospital, Sheffield, S10 2JF UK; 7 Department of Internal Medicine, Cooper University Hospital, Camden, NJ 08103 USA; 8 Royal Stoke University Hospital, Stoke-on-Trent, ST4 6QG UK; 9 School of Medicine, Keele University, Keele, ST5 5BH UK; 10 Department of Internal Medicine, Rutgers Health/Monmouth Medical Center, Long Branch, NJ 07740 USA; 11 Division of Gastroenterology, Hepatology & Nutrition, Brody School of Medicine, Greenville, NC 27834 USA

**Keywords:** Artificial intelligence, Machine learning, Gastroenterology, Hepatology, Public health

## Abstract

**Background:**

Artificial intelligence (AI), which includes machine learning and deep learning, is fundamentally changing public health in gastroenterology and hepatology—fields grappling with a significant global disease burden.

**Objective:**

This review focuses on the population-level applications and impact of AI, highlighting its role in shifting healthcare strategies from reactive treatment to proactive prevention.

**Results:**

AI demonstrates substantial improvements across many different areas. In colorectal cancer, AI models significantly boost detection rates, successfully identifying a large majority of high-risk individuals often missed by traditional screening methods. For metabolic dysfunction-associated steatotic liver disease (MASLD), advanced non-invasive tests offer a high degree of reliability in detecting liver fibrosis. The identification of viral hepatitis is enhanced with excellent accuracy, and gastrointestinal infection surveillance benefits from wastewater analysis that provides an early warning system weeks ahead of clinical case reporting. Furthermore, AI improves the diagnosis of upper GI cancers, such as gastric cancer, with higher diagnostic capability, and facilitates precision public health in inflammatory bowel disease (IBD) through highly accurate risk prediction models.

**Challenges:**

Despite these important advances, significant hurdles remain. Key challenges include ensuring diverse and representative data to prevent algorithmic bias, protecting patient privacy, establishing robust regulatory frameworks for new technologies, and successfully moving innovations from research settings into practical, real-world deployment.

**Conclusion:**

The unequal distribution of AI development and access between high-income countries and low- and middle-income countries risks exacerbating existing health disparities. To fully realize AI's transformative potential for global public health in gastroenterology and hepatology, these cross-cutting issues must be actively addressed through ethical design, rigorous validation, and equitable worldwide deployment.

## Introduction

Gastrointestinal (GI) and hepatological diseases represent a substantial and growing global public health burden, contributing significantly to morbidity, mortality, and healthcare expenditures [1]. Traditional public health interventions, while foundational, often encounter limitations in their scalability, efficiency, and precision when addressing the complex and multifaceted nature of these conditions. The advent of artificial intelligence (AI), encompassing sophisticated methodologies such as machine learning (ML), deep learning (DL), and natural language processing (NLP), presents unparalleled opportunities to revolutionize public health approaches. These technologies can analyze vast and complex datasets, identify intricate patterns, and enabling predictive analytics in ways previously unimaginable [2].

The field of gastroenterology and hepatology is particularly well suited for the application of AI. This specialty heavily relies on various forms of imaging, including endoscopy, radiology, and pathology, which generate enormous volumes of visual data. Additionally, the increasing availability of electronic health records (EHR), multi-omics data (genomics, proteomics, metabolomics), and detailed clinical notes provides a rich, high-dimensional data environment. The sheer volume and complexity of this information, while challenging for conventional epidemiological and analytical methods, constitutes a profound asset for AI transformation. AI’s core strength lies precisely in its ability to process, interpret, and derive meaningful patterns from such “big data,” thereby uncovering previously hidden population health dynamics [3]. This capability positions AI not merely as an incremental improvement but as a fundamental paradigm shift in how public health can leverage existing information to achieve more precise and impactful interventions, making gastroenterology and hepatology a prime candidate for AI-driven public health innovation.

This comprehensive literature review systematically synthesizes the current evidence on AI applications specifically tailored for population-level and public health interventions in gastroenterology and hepatology. It explicitly excludes applications focused solely on individual clinical decision-making. A central objective is to emphasize quantitative data and statistics, including prevalence, incidence, effect sizes, model performance metrics (such as Area under the Receiver Operating Characteristic curve, accuracy, sensitivity, specificity, precision, and recall), screening outcomes, and broader health impact. Furthermore, the review critically addresses pivotal cross-cutting themes: health equity, data privacy and ethics, regulatory frameworks, and implementation science. Global coverage is provided, comparing applications and challenges in high-income countries (HICs) and low- and middle-income countries (LMICs) whenever relevant data are available.

## Applications of AI in GI-Specific Public Health Initiatives

### Colorectal Cancer

Colorectal cancer (CRC) remains a major global public health challenge, ranking as the third most common cancer and the second leading cause of cancer-related mortality worldwide. While population-based screening programs—including fecal tests and colonoscopy—have reduced CRC incidence and mortality over recent decades, persistent challenges remain. These include suboptimal patient adherence, the invasive and costly nature of certain screening modalities, and inequities in access and outcomes. Artificial intelligence (AI) offers promising avenues to enhance CRC control, optimize resource allocation, and improve early detection [4].

AI-driven risk stratification is emerging as a key strategy to personalize and optimize CRC screening. Beyond image-based diagnosis, machine-learning models have been developed using routinely collected electronic health record (EHR) data (age, sex, comorbidities, medications), routine blood tests—particularly complete blood count (CBC) parameters and their longitudinal trends—and stool testing data (e.g., fecal immunochemical test, FIT). These models demonstrate moderate-to-good discrimination, with typical AUC/c-index values of ~ 0.67–0.82. A prominent example is the ColonFlag (also known as MeScore) algorithm, which combines age, sex, and CBC parameters to identify individuals at elevated near-term risk of CRC or advanced precancerous lesions. Validation studies in large US and European cohorts report AUCs of ~ 0.76–0.82, with sensitivity around 63% and specificity ~ 82%, depending on cohort and threshold. While values in the 0.7–0.8 range indicate acceptable discrimination, the key advantage of ColonFlag is its reliance on non-invasive, routinely collected data, making it suitable in settings where colonoscopy is not universally available [5].

Another key contribution comes from Nartowt et al. (2019), who trained and validated an artificial neural network (ANN) on the US National Health Interview Survey (NHIS) dataset using 12–14 self-reportable personal health features (e.g., age, BMI, comorbidities, lifestyle factors). This model achieved a sensitivity of 0.63 ± 0.06, specificity of 0.82 ± 0.04, and a concordance of 0.70 ± 0.02. In stratifying risk, only 6% of CRC cases were misclassified as low risk and 2% of non-CRC cases as high risk, demonstrating the feasibility of AI-driven, self reportable health data for population-level risk stratification [6].

Other approaches have also demonstrated strong performance. In a large multinational dietary study (*n* ≈ 109,343; CRC cases ≈ 7326), Rahman et al. (2023) reported that an ANN trained on dietary and sociodemographic variables misclassified only ~ 1% of CRC cases and 3% of non-CRC cases—implying a sensitivity of ~ 99% and specificity of ~ 97%. Although the precise input variables were not fully detailed, this work highlights the potential of novel, lifestyle-driven risk prediction models to complement existing clinical tools. By contrast, microbiome-derived features (e.g., stool metagenomics) and polygenic risk scores remain at the research stage and are not yet in routine use [7].

Large-scale cohort studies further illustrate the efficiency of AI-based stratification 5. In China, novel ML models designed using the data of 10,874 individuals achieved an AUC of 0.859 in the internal validation cohort, and AUC of 0.888 in the temporal validation cohort [4]. In the United States, an AI model trained on a dataset of across 450,000 individuals aged 45–80 years identified non-traditional CRC risk factors, aiding in improving detection rates from 2.4% (traditional methods) to 12%, with 91% individuals identified to be of higher risk as compared to previously thought [6, 8, 9].

Beyond modeling, AI is also being tested as a tool to address screening inequities. An AI-powered virtual patient navigator, trialed among under-screened patients in New York, doubled colonoscopy completion rates compared with usual care, with high acceptability [10]. During colonoscopy, computer vision–assisted systems for real-time polyp detection have been shown in randomized controlled trials to increase adenoma detection rates by approximately 7–10 absolute percentage points (corresponding to a 20–25% relative improvement) compared with conventional colonoscopy [11], and modeling studies suggest their widespread adoption could further reduce CRC incidence in screened populations [9].

In high-income countries, CRC screening is shifting toward risk-based strategies that incorporate clinical, genetic, and behavioral risk factors to guide the choice of modality. AI can further refine these approaches by integrating multidimensional data, including socioeconomic and longitudinal health records. In LMICs, where colonoscopy capacity is constrained, AI-based stratification may provide a pragmatic means of prioritizing scarce resources, though challenges include limited availability of high-quality local datasets and poor generalizability of Western-trained models. Ongoing initiatives in Asia and Latin America are working to adapt and validate AI tools for diverse populations [12]. Ensuring algorithmic fairness remains critical: models trained on imbalanced datasets risk underestimating risk in certain ethnic or socioeconomic groups [13]. Diverse representation in training cohorts and rigorous bias evaluation are therefore essential to achieving equitable population health benefits.

### Metabolic Dysfunction-Associated Steatotic Liver Disease

Metabolic dysfunction-associated steatotic liver disease (MASLD), formerly known as nonalcoholic fatty liver disease (NAFLD), is an increasingly prevalent global health concern, now affecting over 25–30% of the world’s population, with prevalence projected to rise further amid escalating rates of obesity and type 2 diabetes [14]. The majority of MASLD cases remain asymptomatic until progression to nonalcoholic steatohepatitis, advanced fibrosis, or cirrhosis—stages associated with elevated risks of liver-related morbidity and mortality. Liver fibrosis is the strongest predictor of adverse outcomes in MASLD, underscoring the need for effective population-level strategies for early detection and risk stratification [15]. AI has emerged as a key enabler of non-invasive, scalable solutions for MASLD screening, prognostication, and surveillance.

Traditional screening approaches for MASLD—including liver enzyme panels and ultrasound—suffer from limited sensitivity and specificity, particularly for early-stage disease. AI-powered image analysis offers substantial improvements in this regard. Meta-analyses indicate that AI-assisted imaging models for detecting hepatic steatosis achieve pooled sensitivity in the low 90-percent range (≈ 91-92%) and pooled specificity also in the low 90-percent range (≈ 92-94%), with an AUC of around 0.97 [16]. Similarly, AI applications have enhanced the diagnostic utility of transient elastography (FibroScan) and MRI for assessing steatosis and fibrosis. One ML model employing vibration-controlled transient elastography demonstrated accurate longitudinal risk stratification for liver fibrosis [17]. In a study of NAFLD detection using NHANES 2017-2020 data, an XGBoost AutoML model achieved ≈ 86% AUC, ≈ 79.5% accuracy, ≈ 77.3% sensitivity, and ≈ 80.2% specificity for diagnosing hepatic steatosis compared to controlled attenuation-parameter measurements AutoML models with XGBoost, validation sample: AUC = 0.859; accuracy = 0.795; sensitivity = 0.773; specificity = 0.802 [18].

DL techniques have further advanced non-invasive assessment. For example, Choi et al. applied DL to contrast-enhanced CT (computed tomography) images from over 7000 patients, achieving area under the receiver operating characteristic curve (AUROCs) of 0.95 for cirrhosis (F4), 0.97 for advanced fibrosis (≥ F3), and 0.96 for significant fibrosis (≥ F2). Comparable performance has been demonstrated using ultrasound and CT data in other studies, although variability across cohorts highlights the need for rigorous external validation [19]. Simple yet effective AI models using support vector machines (SVM) applied to routine demographic and biochemical markers have also shown promise. In a Japanese MASLD cohort, an SVM-based model achieved AUROCs of 0.886 for significant fibrosis (≥ F2), 0.882 for advanced fibrosis (≥ F3), and 0.916 for cirrhosis (F4), matching or exceeding conventional non-invasive tests such as Fibrosis-4 Index (FIB-4) and FibroScan-AST (FAST) scores [20, 21]. These models eliminate the need for expensive imaging or specialized biomarkers, increasing feasibility for widespread implementation in primary care settings and community-based screening.

AI models are also being developed to predict progression from early MASLD to advanced fibrosis and cirrhosis, enabling targeted intervention for high-risk individuals. ML models incorporating routine clinical and laboratory data have predicted NASH (biopsy-confirmed) with ~ 86% sensitivity and ~ 81% accuracy. Random forest (RF) models combining common laboratory markers (e.g., AST, platelet count) have outperformed traditional scoring systems in identifying high-risk NASH patients, with AUROCs ~ 0.85 [22, 23]. Further refinement of risk models has been achieved through the integration of insulin-related indices. One study demonstrated that models using homeostasis model assessment of insulin resistance (HOMA-IR), triglyceride glucose-waist circumference index (TyG-WC), age, AST, and ethnicity achieved an AUROC of 0.960. HOMA-IR and TyG-WC consistently emerged as core predictive factors across models [24].

These AI tools are increasingly being deployed in public-facing platforms. For example, the Fatty Liver Foundation in the U.S. offers an online AI-based risk stratification tool enabling individuals to input basic health data and receive guidance on their likelihood of undiagnosed fibrosis, thereby promoting early medical engagement. AI also contributes to surveillance and precision public health efforts in MASLD. ML models applied to longitudinal cohort data have been used to forecast future MASLD incidence in populations, incorporating not only clinical factors but also environmental exposures and behavioral variables [25–27]. This enables public health authorities to identify communities at heightened risk and tailor prevention strategies accordingly.

Moreover, AI is facilitating personalized interventions. Early trials have explored AI-guided lifestyle coaching, where algorithms continuously analyze an individual’s diet, physical activity, and weight trajectory to generate adaptive recommendations aimed at halting or reversing MASLD progression [28]. While still nascent, such approaches offer promise for delivering scalable, tailored preventive interventions. While HICs are progressively integrating fibrosis screening into routine diabetes care, under-diagnosis remains a critical barrier in many LMICs. AI could help bridge this gap by enabling low-cost, scalable screening. For example, DL models capable of analyzing smartphone-acquired ultrasound images could support community-based screening initiatives in rural LMIC settings, where access to specialist radiologists is limited [29].

However, several challenges remain. Most AI models for MASLD have been trained on Western or East Asian populations, raising concerns about generalizability to other contexts due to differences in body composition, comorbidities, and imaging protocols. Developing annotated local datasets and validating models in diverse populations is imperative to avoid introducing bias or misclassification. Cost-effectiveness also warrants careful evaluation. The high sensitivity of many AI-driven fibrosis models may lead to substantial false-positive rates, potentially overwhelming healthcare systems with unnecessary follow-up investigations. Implementation research is needed to optimize tiered screening pathways, ensuring that AI tools enhance rather than strain health service capacity [15].

### Hepatitis

Chronic viral hepatitis—principally Hepatitis B virus (HBV) and Hepatitis C virus (HCV)—remains a major global public health challenge despite the availability of effective vaccines and antiviral therapies. An estimated 296 million people live with HBV and 58 million with HCV worldwide, with viral hepatitis causing ~ 1.5 million deaths annually—a burden comparable to human immunodeficiency virus (HIV) and tuberculosis [26]. A substantial proportion of infections remain undiagnosed; for HBV, only ~ 10% of infected persons globally had been diagnosed as of 2019 [27]. Both the World Health Organization (WHO) and national agencies aim to eliminate viral hepatitis as a public health threat by 2030, with targets including a 90% reduction in new infections and a 65% reduction in mortality from 2015 levels [28]. Achieving these goals requires closing gaps in testing, diagnosis, treatment initiation, and surveillance—domains where AI is emerging as a transformative tool.

AI and ML methods are being deployed to address one of the most formidable challenges in elimination: the large pool of undiagnosed individuals. ML models trained on EHR and claims data in the U.S. have demonstrated strong performance in identifying undiagnosed HCV. Common predictors included age, birth-cohort status (1945–1965), history of injection drug use, opioid or analgesic prescriptions, cirrhosis, HIV/AIDS diagnosis, elevated AST/ALT levels, and comorbidities such as diabetes, hypertension, hyperlipidaemia, depression, and anxiety. Additional demographic factors such as race/ethnicity (notably Black or Hispanic populations) further improved detection [29–32]. In one large study involving 10 million patients, stacked ensemble models achieved 97% precision at > 50% recall, far outperforming logistic regression (31% precision). Similarly, in hemodialysis cohorts, integrating aminotransferase cut-offs with patient age and dialysis duration yielded ~ 97% sensitivity for HCV detection [32].

Evidence from high-risk groups highlights the importance of incorporating biochemical markers into predictive algorithms. In multiple studies of patients with type 2 diabetes, a majority of those with undiagnosed HCV presented with abnormal liver function tests (LFTs). For example, in one cohort of 176 diabetics, 72% of HCV-positive subjects had abnormal LFTs compared with only 25% of HCV-negative individuals [33, 34]. In a multi-ethnic UK cohort, diabetic patients with elevated ALT had significantly higher rates of HCV positivity—most notably among Afro-Caribbean patients, where positivity reached 28% with abnormal ALT versus 4% with normal ALT [35]. Other regional cohorts reported that 65–80% of HCV-positive diabetics had raised ALT, AST, or alkaline phosphatase, though up to 20–35% presented with normal enzyme levels [36]. These findings suggest that while elevated liver enzymes are a strong signal for case detection, relying on LFTs alone risks missing around one in five HCV-positive diabetic patients. AI models trained on broader data domains—including demographics, comorbid conditions, medication history, healthcare utilization, and prescription data—offer the opportunity to enhance sensitivity and ensure fewer cases are missed.

Beyond claims and laboratory data, AI models can incorporate social and behavioral determinants. A LASSO regression model trained on diabetic patients achieved an AUC of 0.81 for predicting HBV/HCV, with illicit drug use, poverty, and race emerging as top predictors [31]. Other models using routine blood panels have classified HBV/HCV infections with very high accuracy. For example, in Kim et al., various machine learning models (including random forest and k-nearest neighbor) applied to NHANES diabetes/hepatitis data achieved 96.75 % overall accuracy [31, 38]. Collectively, these findings highlight AI’s potential for efficient prescreening in both high-risk and general populations.

**Fig. 1:**
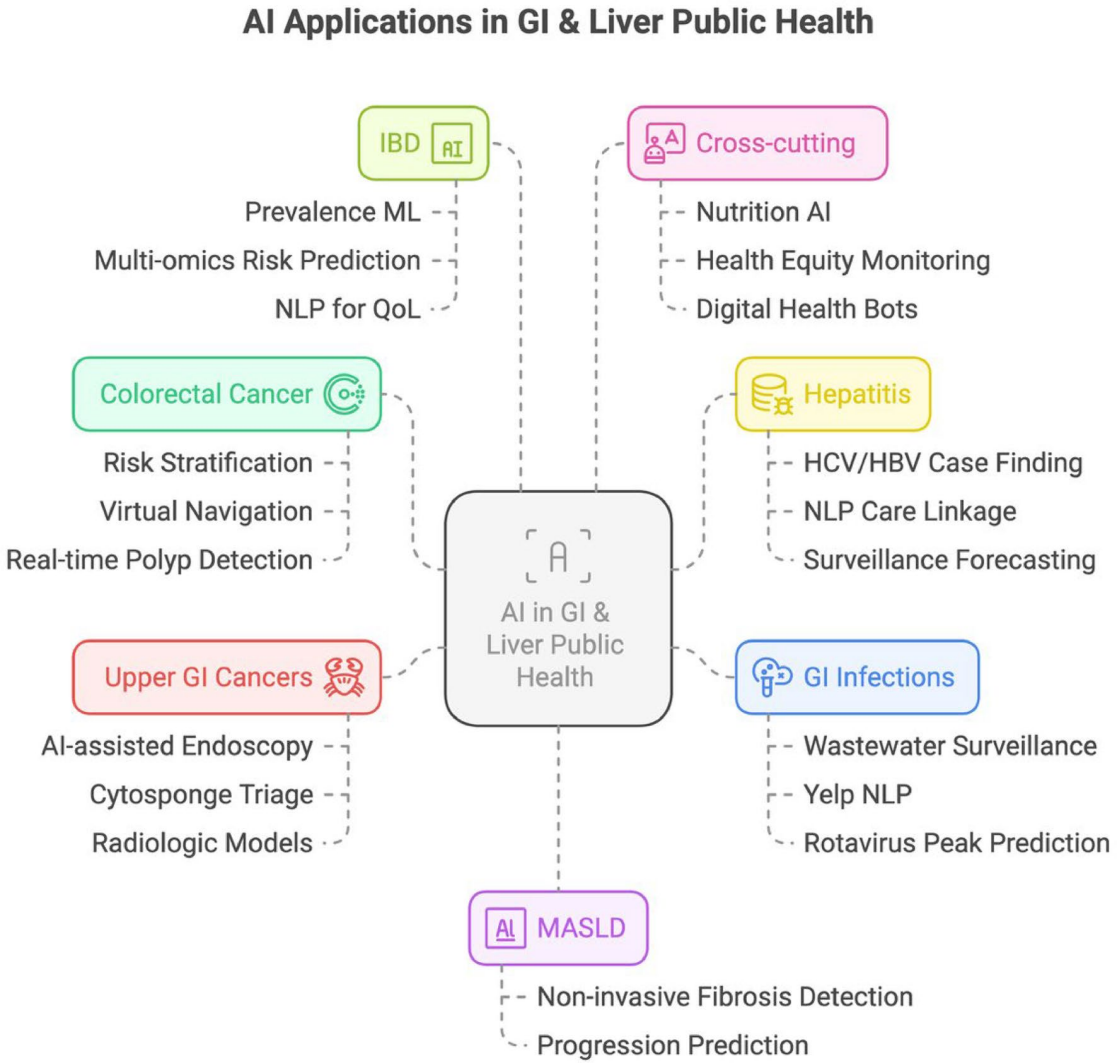
AI Applications in GI & Liver Public Health

AI also improves resource allocation for screening programs, an especially important consideration in LMICs. In a large HCV micro-elimination project in Romania, an ANN trained on risk-factor questionnaires achieved 81.5% sensitivity while recommending ~ 13,400 individuals for testing out of > 15,000 respondents. A more stringent version reduced the testing burden by 60% (to ~ 5200 tests) while still identifying 68% of positives [38, 39]. Such approaches can substantially improve cost-effectiveness and yield, enabling scale-up of hepatitis testing programs (See Fig. 1).

**Fig. 2:**
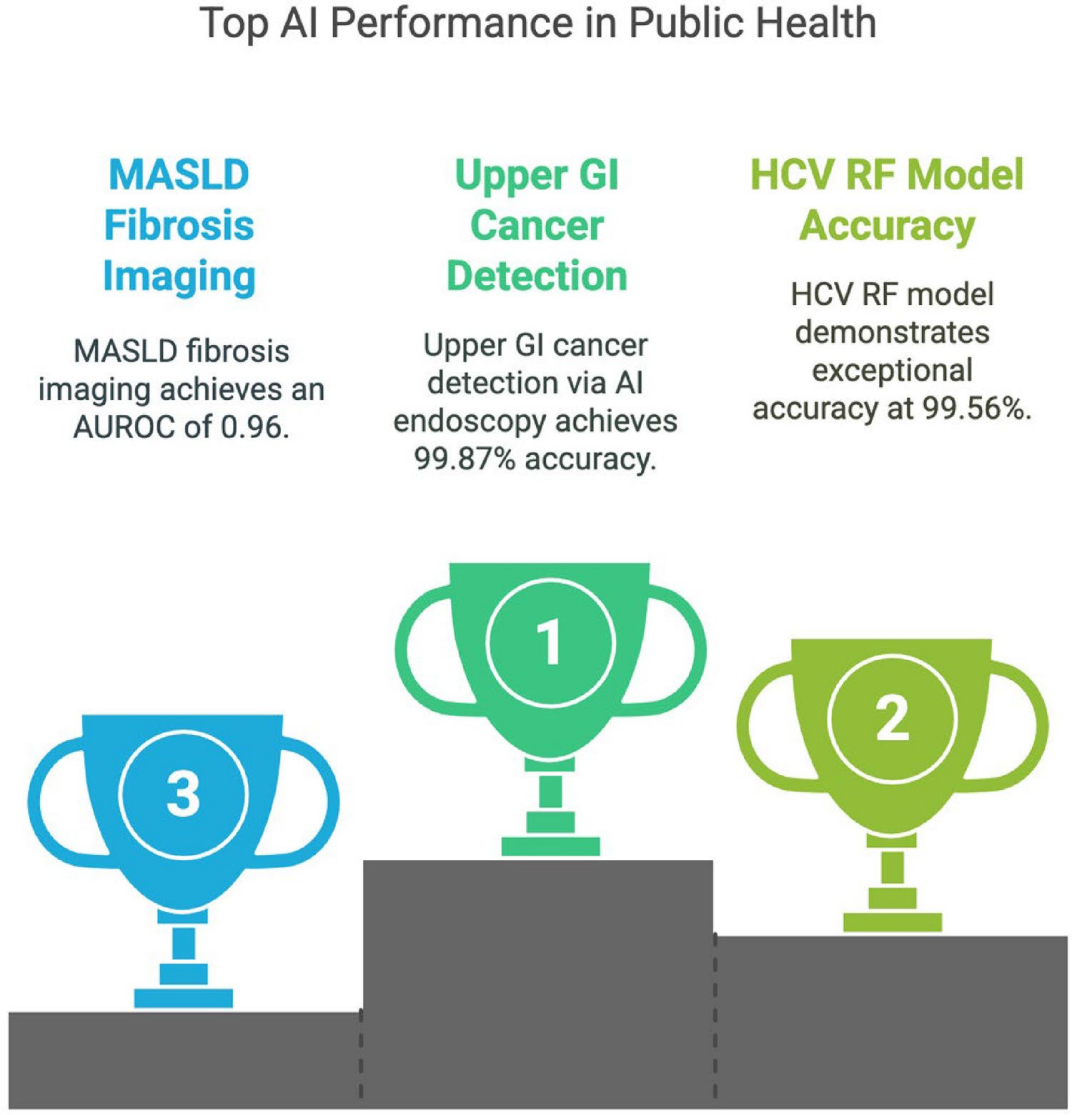
Figure depicts highlights of top AI performance in Public Health GI

Closing the diagnosis-to-treatment gap remains another barrier in the hepatitis care cascade. In the U.S., only 16% of 3.5 million Americans with chronic HCV had received treatment, and only 9% achieved sustained virological response [39]. AI tools are being leveraged to improve linkage to care. In Spain, health authorities used AI-powered text mining of EHRs to flag previously diagnosed HCV patients lost to follow-up, enabling targeted re-engagement [40]. In the U.S., NLP algorithms applied to clinical notes identified HIV/HCV co-infected individuals not receiving therapy, facilitating targeted case management [41]. These interventions directly support WHO elimination goals by improving retention and ensuring timely treatment (See Fig. 2).

At the population level, AI models are increasingly used for surveillance and forecasting. Advanced ML methods such as ANNs and autoregressive integrated moving average (ARIMA) outperform traditional statistical models in predicting incidence trends. For example, ANNs achieved a correlation coefficient of 0.71 for HAV incidence forecasting, compared to 0.66 with ARIMA [30, 31]. In the U.S. and Europe, ML models integrating incidence, treatment uptake, and behavioral data are used to project progress toward 2030 elimination targets. These often reveal major shortfalls, with many countries off-track due to lagging diagnosis and treatment [42]. AI is also being applied to predict long-term outcomes in HBV and HCV patients, including risks of cirrhosis and hepatocellular carcinoma, which can guide surveillance intensity and optimize resource allocation [43, 44].

High-income countries (HICs) have increasingly adopted AI in hepatitis programs. In the U.S., ML models integrated into EHR systems prompt HCV screening in birth cohorts and flag patients at risk of care disengagement [45]. In contrast, most LMICs—despite bearing the greatest HBV burden—are at earlier stages of AI adoption. A key barrier is limited digital infrastructure, particularly lack of robust EHR systems. Nonetheless, early pilots are emerging: in Ethiopia, ML models have been trialed to predict HBV treatment outcomes and inform therapy prioritization [46]. Without technology transfer and capacity-building, however, global disparities in AI readiness risk widening the hepatitis elimination gap. Equity-focused initiatives led by WHO and international partners will be critical to ensure that AI tools are accessible and adapted for resource-limited settings.

### GI Infection Surveillance and Outbreak Prediction

GI infections—caused by pathogens such as norovirus, rotavirus, Salmonella, Vibrio cholerae, and others—continue to pose a major global public health burden. In 2021, diarrheal diseases were responsible for approximately 1.2 million deaths worldwide, including an estimated 390,000 deaths among children under five years of age [47]. The early detection and containment of GI outbreaks are critical for mitigating morbidity and mortality. However, traditional surveillance systems often lag behind the true dynamics of disease transmission and may miss asymptomatic cases that contribute to community spread. AI, particularly when integrated with novel surveillance modalities such as wastewater-based epidemiology (WBE) and digital epidemiology, is driving a transformative shift from reactive to proactive public health responses in GI infection surveillance [48, 49].

Traditional surveillance for GI infections relies heavily on clinician-reported cases and laboratory confirmations, which introduce significant time delays. AI-driven syndromic surveillance can accelerate outbreak detection by analyzing real-time signals from diverse data sources, including emergency department (ED) visit records and symptom query data. For instance, ML models have been successfully applied to ED chief complaint data to identify spikes in GI illness several days before laboratory confirmations. One AI-driven syndromic surveillance model retrospectively demonstrated the capacity to provide earlier alerts for a Campylobacter outbreak compared to conventional reporting systems. In resource-limited settings, simple ML models analyzing trends in clinic visits for diarrhea have been piloted to flag potential Vibrio cholerae outbreaks, enabling timely water safety interventions [51–53].

Advanced time-series algorithms, such as LSTM (long short-term memory) neural networks, have further been employed to forecast seasonal surges in GI infections. For example, LSTM models have successfully predicted rotavirus peak periods, enabling healthcare systems to optimize resource allocation, including stockpiling oral rehydration solutions and preparing clinical capacity for anticipated case loads [52, 53].

**Fig. 3:**
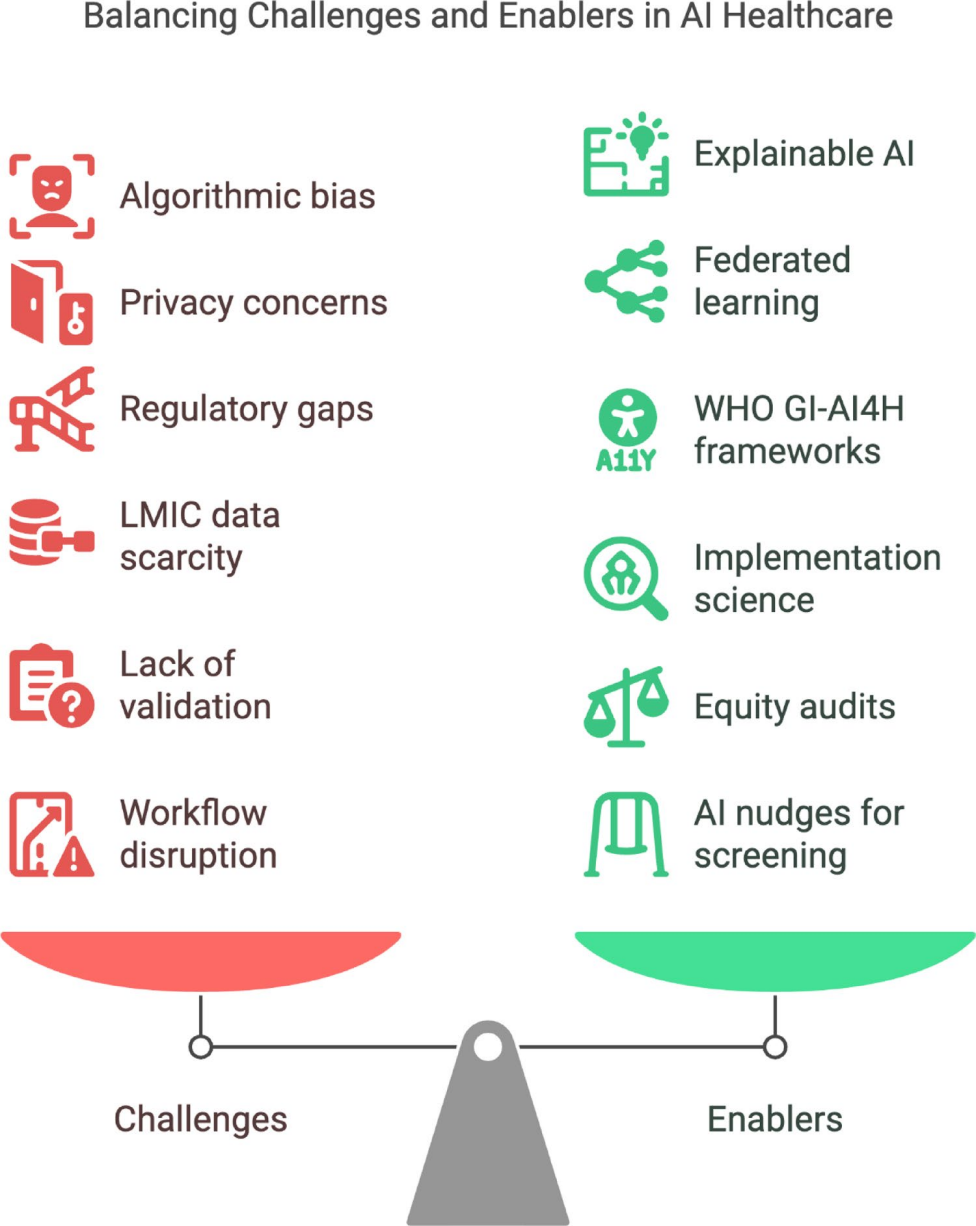
Challenges and Enablers of AI in GI Healthcare

Given that many individuals experiencing GI illness do not seek medical care, non-traditional data sources such as social media and web platforms can provide valuable complementary insights. A landmark initiative by the New York City (NYC) Department of Health demonstrated the utility of mining Yelp restaurant reviews using NLP algorithms to detect foodborne illness signals. In this project, an NLP system scanned approximately 294,000 Yelp reviews over 9 months, identifying 129 potential cases of foodborne illness. Subsequent investigations confirmed three previously undetected restaurant-related outbreaks, none of which had been reported through conventional complaint hotlines [54]. Notably, only 3% of illness incidents flagged via Yelp overlapped with official reports, underscoring the value of AI in capturing signals from otherwise unmonitored population segments. Such approaches are now gaining wider adoption; the UK Health Security Agency is exploring AI-based analysis of online restaurant reviews and social media to enhance foodborne disease surveillance [55]. By automatically classifying text for symptom and food-related mentions, AI augments conventional surveillance systems and increases sensitivity to outbreaks that may otherwise go undetected (See Fig. 3).

WBE has emerged as a powerful tool for early detection of infectious disease outbreaks, including GI infections. WBE involves analyzing wastewater to detect the presence of viral, bacterial, and parasitic pathogens, providing an aggregated, population-level signal that is independent of healthcare-seeking behavior or clinical testing rates. One key advantage of WBE is its capacity to detect pathogens days or weeks before clinical cases surge. For example, in monitoring norovirus GII, wastewater viral levels were found to precede syndromic reports and search term data by 2–3 weeks, offering a valuable lead time for public health response. AI integration further enhances WBE’s predictive capabilities. Hybrid models combining hydraulic simulations of sewer networks with ML (e.g., SVMs) have been used to localize infection hotspots based on spatial patterns of pathogen concentrations in wastewater. During the COVID-19 pandemic, similar approaches were extended to track SARS-CoV-2, and more recently, AI models integrated with automated virus enrichment robots have demonstrated high correlation (87% explained variance) between wastewater monkeypox virus concentrations and clinically confirmed cases [49, 52, 53, 56].

For GI pathogens, ML models have also been used to predict weekly norovirus case counts by learning from wastewater data and environmental factors (e.g., temperature, rainfall), achieving lead times of one week or more ahead of clinical reporting [52, 49, 56]. WBE offers enhanced specificity by detecting pathogen-specific nucleic acids (e.g., HuNoV GII RNA), thereby providing more accurate community infection metrics than general syndromic data alone (See Tables 1 and 2).

**Table 1:** Summary of AI applications in GI-specific population health initiatives

Domain/Disease area	AI applications	Key outputs/impact	Global context/considerations
Colorectal cancer	Risk stratification using ML/ANN; AI virtual navigators for screening adherence; AI-assisted colonoscopy	Improved targeted screening; higher adherence in underserved groups; enhanced adenoma detection	Risk-based screening in HICs; AI to optimize limited resources in LMICs; fairness and generalizability remain challenges
MASLD/NAFLD	AI-enhanced ultrasound, transient elastography, CT/MRI; risk models (SVM, RF); online risk tools	Improved non-invasive screening and fibrosis prediction; personalized risk estimation	HICs integrating fibrosis screening; potential to bridge gaps in LMICs via smartphone-based ultrasound AI
Viral hepatitis (HAV/HBV/HCV)	ML case-finding in EHRs; AI-based risk modeling in high-risk groups; NLP to identify lost-to-follow-up; AI-driven surveillance and forecasting	Increased case detection; improved linkage to care; enhanced forecasting for elimination progress	High adoption in HICs; emerging LMIC pilots; digital infrastructure critical for scaling
GI infection Surveillance	Syndromic surveillance (ED data); WBE + AI; social media/NLP mining; integrated early warning systems	Earlier outbreak detection; predictive modeling of seasonal trends; targeted interventions	HICs leveraging rich data streams; LMICs innovating with satellite, climate, and mobile-based surveillance
Upper GI cancers	AI-assisted endoscopy (CNN, DNN); AI-enhanced sponge cytology; AI on radiologic images; blood-based biomarker models	Improved early lesion detection; prescreening to prioritize endoscopy; reduced inter-observer variability	Pilots in endemic LMIC regions (e.g., Iran, Honduras); generalizability and regulatory challenges remain
Inflammatory bowel disease	Epidemiological modeling; case-finding in administrative data; risk stratification (RF, CDORPF); NLP for burden assessment; multi-omics integration	Enhanced surveillance; improved identification of high-cost patients; insights into patient-experienced burden	Increasing LMIC incidence; AI supports registry building, early detection, and cost-effective care
Dietary interventions & nutritional epidemiology	AI-based dietary assessment tools; personalized nutrition algorithms; ML for dietary pattern analysis	More accurate dietary data; identification of high-risk patterns; precision dietary interventions	Important for public campaigns (CRC, IBD, NAFLD); privacy and technical accuracy remain key concerns
Health inequalities monitoring	ML to identify disparities in care delivery and screening; simulation of intervention equity impacts	Detection of hidden inequities; informs equitable resource allocation	Emphasis on fairness, algorithmic bias testing essential; supports equitable AI deployment globally
Digital health promotion	AI chatbots for vaccination and screening promotion; AI-targeted social media campaigns	Increased outreach and engagement; personalized education	Effective in LMICs; privacy concerns require careful management
GI disease modeling & policy	Agent-based models (e.g., H. pylori); AI-driven economic models for prevention (e.g., liver cancer via aflatoxin reduction)	Informs public health policy; enables scenario testing for interventions	LMICs face data limitations; international collaboration critical for improving model robustness

**Table 2:** Summary of key cross-cutting challenges and mitigation strategies for the equitable, ethical, and effective implementation of AI in population-level gastroenterology and hepatology public health

Theme	Key challenges	Mitigation strategies/best practices
Health equity	Bias from unrepresentative training data- Underperformance in LMICs- Risk of exacerbating existing disparities	Inclusive, representative datasets- Subgroup analyses and debiasing- Community engagement in AI development- International collaboration and adaptation for LMIC contexts
Data privacy and ethics	Privacy risks from large, granular datasets- Inconsistent global data governance- Bias and inequitable model performance- Lack of transparency and explainability- Unclear accountability for AI-driven errors	Federated learning and privacy-preserving techniques- Explainable AI- Mandating algorithmic transparency- Clear accountability pathways- Complementing, not replacing, clinical judgment
Regulatory frameworks	Fragmented oversight, especially for population-level AI tools- Lack of clear validation and monitoring standards- Uncertainty around accountability in public health AI applications	Harmonized cross-border AI governance- Lifecycle assessment of AI tools- Transparency mandates (e.g., EU AI Act)- Promoting clinician and public health AI literacy
Implementation science	Gap between high-performing AI models and real-world adoption- Data generalizability concerns- Black-box models undermine trust- Infrastructure and cost barriers- Workflow integration challenges	Multidisciplinary collaboration- User-centered design and iterative improvement- Prospective real-world validation- Behavioral interventions (e.g., digital nudges)- Focus on human and organizational factors for successful adoption

State-of-the-art GI outbreak prediction systems increasingly leverage data fusion—integrating clinical, environmental, digital, and laboratory data streams—via AI to generate comprehensive early warnings. Techniques such as Bayesian networks and DL architectures enable the combination of heterogeneous inputs to improve outbreak detection accuracy. For example, an early warning system under development by the European Centre for Disease Prevention and Control integrates hospital admissions, Google Trends (searches for terms like “vomiting”), weather data, and news feeds to compute real-time outbreak probabilities for various GI pathogens. Preliminary results demonstrate improved sensitivity relative to any single data source, although false-positive rates remain a challenge. Moreover, NLP techniques are being used to parse social media posts and news articles to detect emerging GI outbreaks—such as norovirus outbreaks on cruise ships—sometimes before formal notifications reach public health authorities [8, 51–56]. The fusion of WBE, clinical data, and digital epidemiology enables a more proactive approach to outbreak management.

HICs benefit from rich data ecosystems (e.g., widespread internet access, comprehensive EHRs) that facilitate advanced AI-driven GI surveillance. However, LMICs, where GI infections such as cholera and dysentery are endemic, are also adopting innovative AI approaches. In Bangladesh, ML models utilizing satellite-derived river height, rainfall, and temperature data have been used to accurately predict cholera outbreaks, guiding targeted vaccination campaigns [57]. Mobile phone-based reporting systems, combined with AI, are also being piloted in LMIC settings to crowdsource symptom data for real-time outbreak detection [58]. Nevertheless, important equity considerations must be addressed. Heavy reliance on digital data streams (e.g., social media, smartphones) may systematically underrepresent rural or low-income populations. The NYC Yelp project demonstrated that AI-based surveillance captured a demographically distinct population compared to traditional complaint systems [54]. To ensure inclusive surveillance, public health agencies must deploy multiple complementary AI models tailored to different population segments. Moreover, privacy concerns related to mining personal data must be carefully managed through transparent governance frameworks that balance public health benefits with individual rights.

### Upper GI Cancers (Gastric and Esophageal)

Cancers of the upper GI tract—principally gastric cancer and oesophageal cancer—remain major global contributors to cancer morbidity and mortality. Gastric cancer is the fifth most common malignancy worldwide, with over 1 million new cases and nearly 770,000 deaths in 2020, and exhibits high incidence in East Asia, parts of Latin America, and Eastern Europe. Oesophageal cancer, with approximately 0.6 million new cases in 2020, is highly lethal, with two dominant histological subtypes: squamous cell carcinoma (ESCC), prevalent in East Asia and Eastern Africa, and adenocarcinoma, more common in Western countries [59]. Early detection is paramount to reducing mortality, as late-stage diagnosis is common due to vague early symptoms. However, conventional screening methods, including endoscopy, remain invasive, costly, and dependent on specialist expertise—barriers that limit participation and scalability, particularly in resource-constrained settings. In this context, AI is emerging as a transformative tool to enhance the effectiveness, accessibility, and efficiency of upper GI cancer screening and surveillance.

AI-driven computer vision models—particularly convolutional neural networks (CNNs) and deep neural networks (DNNs)—have demonstrated substantial potential in augmenting endoscopic detection of early-stage gastric cancer and ESCC [60]. AI-assisted white-light imaging endoscopy has reported diagnostic accuracy exceeding that of human experts. For example, one gastric cancer model achieved a per-image detection rate of 99.87%, compared with 88.2% for expert endoscopists. Similarly, AI-assisted systems for early gastric cancer have demonstrated detection rates of ~ 93.2% [60]. In oesophageal cancer screening, a DNN trained on 2428 endoscopic images achieved 97.8% sensitivity, 85.4% specificity, and 91.4% overall accuracy for detecting ESCC—substantially outperforming both senior (88.8%) and junior endoscopists (77.2%) [61, 62]. A CNN trained on 6,473 narrow-band imaging endoscopic images for early dysplasia and ESCC achieved 98% sensitivity and 95% specificity [62]. Importantly, AI assistance has been shown to significantly improve endoscopist performance, especially among less experienced clinicians, while reducing inter-observer variability and promoting more standardized diagnostic quality across healthcare settings.

Despite these promising results, several limitations must be acknowledged. The exceptionally high detection rates often reported arise from retrospective studies using enriched image datasets under controlled conditions, which may not reflect real-world practice. Performance typically degrades in external or prospective validation, with significant drops in specificity and positive predictive value (PPV), raising concerns about false-positive findings and overcalling of lesions. For example, broader evaluations of AI-assisted gastric cancer detection reported pooled sensitivity of ≈ 86% and specificity of ≈ 93%, indicating strong but not perfect precision [63]. These findings suggest that while AI can substantially reduce missed lesions, it should currently be regarded as a supplementary tool rather than a replacement for real-time expert judgment. Future work must focus on prospective validation, improving specificity and PPV, and testing across diverse clinical settings.

Deployment in high-incidence regions is already demonstrating tangible benefits. In China, mass screening programs for oesophageal and gastric cancer have integrated AI-based image analysis to improve lesion detection and workflow efficiency. One CNN trained on ~ 8400 oesophageal cancer images detected lesions < 1 cm in size—frequently missed by human observers—processing > 1100 test images in just 27 s [60, 95]. Similarly, population-based screening programs in China have shown that AI integration improves early ESCC detection, thereby enhancing curability.

AI is also being applied to alternative, less invasive modalities in settings where endoscopy is not widely feasible. For oesophageal cancer, sponge cytology (e.g., Cytosponge) analyzed with ML algorithms has achieved > 90% specificity for distinguishing high-grade dysplasia from benign changes, supporting its role as a triage tool for endoscopy referrals [4, 98]. In radiologic screening, DL models based on Faster R-CNN applied to barium meal studies increased diagnostic accuracy from 89.3% to 96.8% while reducing radiologist interpretation time. CNN-based models analyzing computed tomography images for oesophageal cancer screening have achieved average accuracies of 86.4% ± 5.6% [61, 100]. Gradient boosting models combining non-invasive features such as *Helicobacter pylori* infection status, blood markers, and demographic data have also demonstrated promising results [4, 61, 99].

In endemic regions such as the “oesophageal cancer belt” (East Africa to Central Asia) and high gastric cancer prevalence areas (e.g., Andean South America), AI-supported screening is being piloted to extend specialist capacity. In Iran, AI-assisted minimally invasive endoscopic programs have improved ESCC lesion detection and are being scaled across rural provinces [64]. In Honduras, portable AI-enhanced endoscopy is enabling non-specialists to perform gastric cancer screening in remote areas, with AI guidance improving accuracy [65, 66]. These examples illustrate the potential of AI to support task-shifting, empowering allied health personnel to deliver effective screening in contexts where gastroenterologists are scarce.

High-income countries (HICs) with established screening programs, such as Japan, are at the forefront of integrating AI into endoscopy for workflow efficiency and quality improvement [67, 68]. In contrast, most low- and middle-income countries (LMICs) have yet to implement large-scale upper GI cancer screening due to financial and infrastructural constraints. AI cannot overcome fundamental limitations in endoscopic capacity but can increase the efficiency and public health impact of limited programs. Key challenges for broader implementation include ensuring external validation across diverse populations—since most models are trained on East Asian datasets with limited generalizability—alongside regulatory approval, clinical safety monitoring, and cost-effectiveness evaluation [69, 70]. Economic modeling suggests that even modest improvements in early detection could make AI-assisted endoscopy cost-effective, given the high costs of late-stage cancer care [9]. Moreover, workflow optimization, reduced repeat procedures, and AI-guided non-specialist screening could further offset costs.

### Inflammatory Bowel Disease

Inflammatory bowel disease (IBD), comprising Crohn’s disease (CD) and ulcerative colitis (UC), represents an escalating global public health challenge. Historically concentrated in Western nations, IBD incidence and prevalence have risen sharply in newly industrialized regions since the late twentieth century, driven by urbanization, Westernized diets, and changing environmental exposures [71]. Globally, the prevalence of IBD increased from approximately 3.3 million cases in 1990 to nearly 6.8 million cases by 2019 [72]. In one cohort, 2021 prevalence reached 218.3 cases per 100,000 people (77.2 for CD, 141.1 for UC), with incidence trends continuing to rise steadily [73, 74]. This epidemiological shift underscores the need for global health systems—including those in LMICs—to prepare for increasing IBD burden. AI is emerging as a vital tool across multiple dimensions of IBD public health: refining epidemiological understanding, improving case detection and risk stratification, quantifying broader societal burden, and advancing precision prevention research.

AI is enhancing the granularity and accuracy of global IBD surveillance. ML has been applied to model incidence patterns across geography and time, supporting proactive public health planning. A notable effort by the Global IBD Collaborative used ML clustering to categorize countries into four “epidemiologic stages” of IBD emergence and spread, identifying regions such as parts of South Asia and Africa where incidence is now rising and public health infrastructure must be prepared accordingly. The ML-driven analysis confirmed that societal westernization consistently precedes IBD emergence, informing policy planning on specialist training and care capacity expansion [71, 73, 74]. Administrative data analyses using AI also offer robust prevalence estimates. In the Netherlands, a RF model achieved an AUROC of 0.97 for identifying IBD cases in health records, with a prevalence of 577.6 per 100,000 and an incidence of 20.1 per 100,000 in 2020 [75]. In the U.S., between 2001 and 2018, IBD prevalence rose with an annual percentage change (APC) of 3.4% for CD and 2.8% for UC, with disproportionate increases among non-Hispanic Black populations (CD APC = 5.0%, UC APC = 3.5%) [73, 80]. These findings, powered by AI, refine our understanding of evolving IBD epidemiology and help guide resource allocation.

Although classic screening programs do not exist for IBD, AI-driven tools can assist in case-finding and early intervention. AI also supports risk stratification among diagnosed patients. ML models have been used to predict healthcare utilization, identifying the subset (~ 20%) of IBD patients likely to drive 80% of healthcare costs through hospitalizations or surgeries [76]. Public health programs can leverage such models to prioritize intensive interventions (e.g., early biologic therapy, nurse-led care pathways) for high-risk patients, improving outcomes and optimizing resource use. Furthermore, AI models predicting premature mortality in IBD have demonstrated AUROCs of 0.81–0.95, supporting population-level risk assessment [77].

Beyond clinical endpoints, AI techniques enrich our understanding of IBD’s broader societal impacts. NLP has been used to analyze social media content and online patient forums, providing real-time insights into quality of life and unmet needs. For example, sentiment analysis of thousands of Reddit posts from IBD communities revealed peaks in negative sentiment correlating with known disease activity trends, highlighting the potential of digital epidemiology to complement traditional surveillance [78]. AI models applied to insurance claims data further elucidate the economic burden of IBD, identifying key cost drivers and informing policy interventions. Such analyses support decisions around subsidizing medications or investing in mental health support, aiming to mitigate indirect burdens such as work disability and reduced quality of life [79, 80].

Although IBD prevention remains complex due to its multifactorial etiology, Geospatial AI analyses have identified IBD incidence clusters potentially linked to environmental exposures such as pollution or climate factors, warranting further investigation [81]. AI’s capacity to integrate multi-omics data—encompassing genomics, transcriptomics, proteomics, metabolomics, and the microbiome—further advances understanding of IBD pathogenesis and progression. The Comprehensive Data Optimization and Risk Prediction Framework (CDORPF), an ensemble ML model trained on gut microbiome data, achieved classification accuracy, recall, and F1 scores exceeding 0.9 for IBD risk prediction [82–84]. A RF model based on laboratory markers attained AUROCs of 97% for CD and 91% for UC [85]. Large Language Models (LLMs) are also transforming population-level IBD research. Fine-tuned LLMs have demonstrated high performance (F1 score improved from 0.7 to 0.82) in structuring unstructured histology and radiology reports from EHRs, unlocking vast real-world data for epidemiological analysis [84]. This capability accelerates research and supports privacy-compliant, large-scale AI-driven public health solutions.

AI’s role in IBD public health varies by region. In HICs, where IBD care is advanced but costly, AI often enhances efficiency—for example, predicting which patients may avoid hospitalization or tailoring biologic therapy. In LMICs, where IBD awareness and diagnostic capacity may be limited, AI can help “put IBD on the map” by supporting case-finding and building virtual disease registries from fragmented health data. International collaborations, such as the Global IBD Visualization Project, are exploring federated learning approaches to enable AI training across multinational datasets without compromising data privacy [86]. This ensures that AI models become globally representative and equitable. However, careful attention to data privacy, algorithmic bias, and model generalizability is paramount, particularly when leveraging sensitive multi-omics datasets [83]. Equity concerns must also be addressed; as advanced analytics improve IBD care in well-resourced centers, efforts must be made to extend these benefits to underserved populations. Without intentional policy and implementation efforts, there is a risk of an “AI gap” in IBD public health.

### Other Public Health Applications in Gastroenterology

Beyond specific disease-focused applications, AI is driving innovation across several cross-cutting domains in GI public health. These include dietary interventions and nutritional epidemiology, health inequalities monitoring, digital health promotion, and disease modeling for public health policy. Together, these applications demonstrate AI’s expanding role in precision public health, enabling more personalized, efficient, and equitable GI health interventions.

## Dietary Interventions and Nutritional Epidemiology

Diet is a critical modifiable risk factor across a spectrum of GI diseases, including CRC, IBD, IBS, and NAFLD. However, conventional dietary assessment methods—such as food frequency questionnaires and 24-h dietary recalls—are labor-intensive, prone to recall bias, and of limited utility for large-scale, accurate nutritional surveillance.

AI-assisted dietary assessment tools, integrated into mobile or web-based platforms, are transforming this landscape. These tools are broadly categorized as image-based (leveraging computer vision for food recognition and nutrient estimation) and motion sensor-based (analyzing wrist movement, jaw motion, and eating sounds to capture eating occasions). Image-based tools can provide real-time, objective dietary data, substantially reducing recall and reporting biases. For example, the SNAQ app demonstrated slightly higher agreement with doubly labeled water (DLW), the gold standard for energy intake measurement, than conventional 24-h recall. Across studies, AI-based dietary tools report accuracy ranging from 60 to 95%, making them viable for both clinical and population-level applications [87].

AI also enhances the granularity of nutritional epidemiology. Traditional analyses often focus on single nutrients; AI enables the analysis of complex dietary patterns and their associations with GI diseases. For example, ML has been used to identify dietary patterns that correlate with higher CRC risk in large cohorts—one study demonstrated that high processed meat intake combined with low fiber intake predicted CRC risk better than any single nutrient [7, 88]. These insights inform public health dietary guidelines.

At an individual level, AI is advancing precision nutrition. In a randomized controlled trial for IBS, an AI-based personalized diet intervention, which used microbiome and dietary preference data to tailor recommendations, led to significantly improved gut symptom scores compared to standard dietary advice [87, 90]. Furthermore, AI tools analyzing genomic, metabolomic, and microbiome data are being explored to guide dietary interventions aimed at enhancing cognitive performance and mitigating gut–brain axis-related symptoms—highlighting the relevance of diet to both GI and neurological health [89].

The ability of AI-assisted tools to deliver real-time, objective dietary data at scale addresses a critical data gap in public health nutrition. This enables more precise identification of dietary patterns linked to GI outcomes, supports targeted health promotion campaigns, and facilitates personalized nutritional guidance. However, technical challenges such as portion size estimation and data privacy concerns require ongoing research and governance [90].

## Health Inequalities Monitoring

AI offers powerful tools to reveal and address disparities in GI health outcomes and care delivery. By mining large-scale health data, ML algorithms can identify geographic, racial, and socioeconomic patterns in disease burden and healthcare access. For instance, a study applying ML to U.S. EHR data found that African American IBD patients were less likely to receive high-quality care measures—an inequity associated with higher hospitalization rates [5, 91, 92]. Similarly, predictive analytics in CRC screening have been used to map neighborhoods with low screening uptake based on social determinants, enabling targeted deployment of mobile screening units.

AI can also model the equity impacts of interventions. One simulation study demonstrated that if an AI-based CRC risk stratification tool systematically underestimated risk in minority populations due to biased training data, it could exacerbate health disparities—prompting developers to adjust algorithms to ensure fairness. Consequently, the GI community is actively developing frameworks for ethical AI deployment that mandate testing for algorithmic bias and representation of diverse populations [91].

## Digital Health Promotion

AI is enhancing digital health promotion in GI by enabling scalable, personalized communication. Chatbots powered by NLP are being used to educate the public about preventive measures such as hepatitis vaccination and colonoscopy preparation. In Latin America, a bilingual AI chatbot promoting HBV vaccination engaged tens of thousands of young adults at a fraction of the cost of traditional campaigns [92, 93].

AI also enables targeted health messaging via social media. Public health agencies can use algorithms to identify users at elevated risk (e.g., members of heavy alcohol use forums who might benefit from information on alcohol-related liver disease) and deliver tailored educational content. While this approach can amplify reach and engagement, it raises privacy considerations that must be carefully managed to maintain public trust [93, 94].

## GI Disease Modeling and Policy

AI-driven simulation models are increasingly informing public health policy decisions in GI. Agent-based models, for example, have been used to simulate HP transmission dynamics in communities, allowing policymakers to test the potential impact of interventions such as mass antibiotic treatment on future gastric cancer incidence. Similarly, AI-driven economic models have evaluated interventions such as food fortification with micronutrients to prevent liver cancer by reducing aflatoxin exposure. These models integrate complex, multifactorial data—including epidemiological, behavioral, and economic inputs—into actionable insights for resource allocation. However, the accuracy of such models is highly dependent on data quality, which remains a challenge in LMIC settings where data may be sparse or fragmented. International data-sharing initiatives and collaborative modeling efforts are essential to improve the robustness of AI-driven policy simulations.

## Cross-Cutting Themes: Equity, Privacy, Regulation, and Implementation

AI offers transformative opportunities for public health applications in gastroenterology and hepatology, yet it also raises several complex cross-cutting challenges that transcend disease-specific domains. Key considerations include health equity, data privacy and ethics, regulatory oversight, and the practical implementation of AI in real-world healthcare settings. These factors must be addressed thoughtfully to ensure that AI-driven innovations ultimately improve health outcomes in an equitable, trustworthy, and sustainable manner.

## Health Equity

AI has the potential to either exacerbate or ameliorate existing health disparities. The outcome depends critically on how AI tools are designed, validated, and deployed. Unrepresentative training data can perpetuate bias; for example, a CRC risk model trained primarily on European populations may underperform when applied to African or Asian populations. Furthermore, algorithmic design choices may inadvertently amplify inequities if they overweight factors correlated with socioeconomic status or structural determinants of health. To mitigate these risks, several best practices are being adopted across the field. These include incorporating race and ethnicity as model inputs where appropriate, conducting rigorous subgroup analyses, applying debiasing techniques, and ensuring that AI development teams are diverse and inclusive [91]. Community engagement is increasingly recognized as essential; the meaningful involvement of underserved populations in AI tool development can help identify and address potential inequitable impacts early. In gastroenterology, frameworks have been proposed to institutionalize practices such as bias audits, algorithmic transparency, and diversity in research cohorts. Addressing global disparities in AI development and deployment is also crucial. The majority of AI studies in GI and hepatology to date have been conducted in HICs, with relatively few originating from LMICs. Without deliberate adaptation, AI models developed in HICs may not generalize well to LMIC contexts. Promoting AI literacy, ensuring inclusive data collection, and fostering international collaborations are critical to achieving the WHO vision of an AI ecosystem that advances health equity and the Sustainable Development Goals. The overarching goal is not simply to create powerful AI tools, but to do so in a way that leaves no population behind [7, 91].

## Data Privacy and Ethics

AI applications in GI public health rely heavily on large datasets, including EHRs, imaging, genomics, and, increasingly, non-traditional sources such as social media or mobile phone data. This reliance raises significant concerns around data privacy, security, and informed consent. Anonymization techniques are essential but imperfect; as datasets grow in size and granularity, the risk of patient re-identification increases [95]. Furthermore, global variation in data protection standards—such as HIPAA (Health Insurance Portability and Accountability Act) in the U.S. and GDPR (General Data Protection Regulation) in Europe—creates inconsistencies in data governance [96]. Particularly concerning are novel data streams, such as internet searches or phone metadata used for outbreak prediction, which often fall outside established regulatory frameworks. Algorithmic bias represents another critical ethical concern. AI models trained on skewed datasets can exhibit differential performance across demographic groups, compounding health inequities. In gastroenterology and hepatology, where disease patterns vary significantly across ethnic and geographic populations, ensuring that AI models perform equitably across diverse groups is paramount. Transparency and explainability of AI systems are also essential for ethical deployment. The “black-box” nature of many DL models impedes trust and informed consent; patients must understand how AI influences their care and have confidence that its outputs are interpretable and accountable. Moreover, legal responsibility for AI-driven errors remains poorly defined, raising challenging questions of liability [97]. To address these concerns, federated learning is being explored to enable collaborative model training without centralizing sensitive data, while privacy-preserving techniques such as differential privacy and homomorphic encryption further enhance data protection. Promoting explainable AI is vital to build clinician and patient trust, and regulatory guidelines should mandate algorithmic transparency and clearly defined accountability pathways. Ultimately, AI must complement—not replace—clinical judgment, preserving the central role of the physician–patient relationship.

## Regulatory Frameworks

The regulatory landscape for AI in healthcare remains fragmented and underdeveloped. While regulatory agencies such as the U.S. Food and Drug Administration (FDA) and European authorities have approved clinical AI devices, such as AI-based polyp detection in colonoscopy, oversight for population-level health applications is less defined. Tools used for screening prioritization or outbreak prediction often lack clear regulatory pathways, creating uncertainty regarding validation standards, accountability, and performance monitoring. For example, if a public health department employs an AI model to prioritize CRC screening for certain groups, it remains unclear who is accountable if the model underperforms or introduces bias. International initiatives are beginning to address these gaps. The WHO’s Global Initiative on AI for Health (GI-AI4H) emphasizes governance, transparency, and ethical principles for the adoption of AI in public health. Similarly, the European Union’s AI Act mandates transparency regarding data provenance and model scope—an approach that could serve as a model for global harmonization. Moving forward, harmonizing cross-border data sharing and AI oversight, adopting risk-based approaches and lifecycle assessments for AI tools, mandating transparency and data quality standards, and promoting equitable infrastructure access will be essential. Additionally, educating clinicians and public health professionals about AI will equip them with the skills needed to critically evaluate and safely integrate AI tools [92, 94, 96, 98].

## Implementation Science

Perhaps the most underappreciated challenge in AI-driven GI public health is the translation of promising models into real-world impact. Many high-performing AI models remain confined to academic publications, with limited uptake in clinical or public health practice. Implementation science provides valuable frameworks to address this gap. Key barriers include data quality and generalizability, as many AI models are trained on small, retrospective datasets that may not generalize to diverse real-world populations; lack of explainability, which undermines clinician trust; unresolved ethical and legal concerns around accountability; high infrastructure costs; and poor integration with existing clinical workflows. Overcoming these barriers requires multidisciplinary collaboration, user-centered design, and rigorous prospective validation. Behavioral interventions—such as digital nudges—offer a promising pathway for promoting AI-driven public health behaviors. Digital nudges have been shown to significantly improve cancer screening adherence, highlighting that human factors such as engagement, usability, and clinician buy-in are as critical as technical performance. Successful implementation examples, such as AI-driven HCV screening in U.S. community clinics, underscore the importance of iterative design and user training. In one such case, initial resistance due to poorly designed alerts and perceived workflow disruption was mitigated through redesign and staff education, leading to improved uptake and testing rates. Ultimately, moving AI from bench to public health impact requires robust pilot programs, real-world validation, and a deep focus on the human and organizational factors that shape healthcare delivery. Without such efforts, even the most sophisticated AI models will fail to achieve their potential to improve population GI and liver health [99–102].

## Discussion

The evidence presented in this comprehensive review underscores the transformative potential of AI in advancing population-level gastroenterology and hepatology public health goals. AI is fundamentally reshaping approaches to disease surveillance, screening, risk stratification, and intervention optimization, enabling a crucial shift from reactive to proactive public health strategies.

Across diverse disease areas, AI has demonstrated consistent and measurable quantitative improvements. In CRC, AI models have significantly increased detection rates by identifying non-traditional risk factors, potentially predicting thousands of future cases and optimizing screening resource allocation [103]. For MASLD, AI-developed non-invasive tests show high diagnostic accuracy and sensitivity for fibrosis, with optimal predictive models achieving AUROCs as high as 0.960 [104]. In viral hepatitis, AI models have achieved exceptional precision (up to 97%) and accuracy (up to 99.56%) in identifying undiagnosed populations, offering a powerful tool to accelerate progress toward global elimination targets GI infection surveillance is being revolutionized by AI’s integration with WBE, providing early warning signals weeks before clinical cases emerge and capturing a high percentage of outbreak variability [49, 50, 53]. For upper GI cancers, AI-assisted endoscopy and non-invasive methods have shown superior diagnostic rates and accuracy, even outperforming human experts, thereby democratizing early cancer detection, particularly in resource-limited settings [60, 61]. In IBD, AI is enabling precision public health by analyzing complex multi-omics data and predicting risk with high AUROCs (up to 0.97), moving beyond broad epidemiological trends to granular, data-driven strategies. Furthermore, AI-assisted dietary assessment tools are bridging critical data gaps in nutritional epidemiology, providing real-time, objective dietary data with high accuracy, enabling more precise population-level nutritional interventions.

The synergistic potential of combining different AI technologies—such as ML for risk stratification, NLP for extracting insights from EHRs, and DL for advanced image analysis—is immense. When integrated with novel data sources like WBE, these combinations create powerful tools for comprehensive population health monitoring and intervention.

However, realizing AI’s full transformative potential in population GI health requires navigating significant overarching challenges. Systemic issues related to data quality, algorithmic bias, patient privacy, and the “black-box” nature of many AI models remain substantial hurdles. The “pacing gap” between the rapid advancement of AI technology and the slower development of adequate legal and regulatory frameworks is a major constraint, hindering widespread, ethical, and safe deployment [97]. Furthermore, the observed disparities in AI research and implementation, predominantly concentrated in HICs with limited representation from LMICs, pose a significant threat to global health equity. If not addressed proactively, AI could inadvertently amplify existing health inequalities by creating solutions that are not generalizable or accessible to underserved populations [1].

Despite these challenges, significant opportunities exist. AI can bridge existing resource gaps in healthcare, democratize access to specialized diagnostics, and enable personalized public health interventions at scale, particularly in regions with limited medical infrastructure. The increasing availability of large, diverse datasets (e.g., through EHRs, multi-omics initiatives) and ongoing advancements in explainable AI offer promising pathways to overcome current limitations.

While AI demonstrates significant promise in improving detection and risk stratification, its ultimate impact on population-level morbidity and mortality—such as a measurable reduction in disease incidence, improved survival rates, or decreased hospitalization rates—requires long-term, prospective studies and successful implementation science. The measurable benefits, such as increased detection rates and improved diagnostic accuracy, are crucial steps, but their translation into tangible health outcomes at the population level is the ultimate measure of success. The observed disparity in AI research and implementation, predominantly in HICs, with a notable exception of China, highlights the critical need for equitable development and deployment of AI solutions tailored to the unique challenges and resource constraints of LMICs. International collaborations and knowledge sharing are paramount to ensure that AI benefits all populations, not just those in well-resourced settings.

## Conclusion

AI is unequivocally poised to become an indispensable tool for advancing population-level gastroenterology and hepatology public health goals. Its unparalleled capabilities for enhanced surveillance, precise screening, targeted risk stratification, and optimized intervention strategies offer a transformative pathway to better health outcomes globally.

To fully realize AI’s profound potential, concerted efforts are imperative from researchers, policymakers, healthcare systems, and industry stakeholders. These efforts must address critical challenges related to data ethics, ensuring privacy and mitigating algorithmic bias through diverse and representative datasets. Regulatory harmonization across international borders is crucial to provide a clear, consistent framework for AI development and deployment, fostering innovation while safeguarding public trust and accountability. Furthermore, equitable access to AI technologies must be prioritized, particularly for LMICs and underserved communities, to prevent the exacerbation of existing health disparities. Future endeavors must emphasize interdisciplinary collaboration, robust real-world validation through implementation science, and a steadfast commitment to health equity, ensuring that AI serves as a powerful force for a more just and healthier world for all.

## Data Availability

Data availability is not applicable to this article as no new data were created or analyzed in this study.

## Abbreviations

GI: Gastrointestinal
AI: Artificial intelligence
ML: Machine learning
DL: Deep learning
NLP: Natural language processing
EHR: Electronic health records
HICs: High-income countries
LMICs: Low-income countries
CRC: Colorectal cancer
NHIS: National health interview survey
PLCO: Prostate, lung, colorectal, and ovarian cancer screening trial
ANN: Artificial neural networks
MASLD: Metabolic dysfunction-associated steatotic liver disease
NAFLD: Nonalcoholic fatty liver disease
NHANES: National health and nutrition examination survey
AUROC: Area under the receiver operating characteristic curve
SVM: Support vector machine
FIB-4: Fibrosis-4 index
FAST: FibroScan-AST
CT: Computed tomography
HOMA-IR: Homeostasis model assessment of insulin resistance
TyG-WC: Triglyceride glucose-waist circumference index
U.S: United States
HAV: Hepatitis A virus
HBV: Hepatitis B virus
HCV: Hepatitis C virus
HIV: Human immunodeficiency virus
RF: Random forest
WHO: World health organization
LASSO: Least absolute shrinkage and selection operator
ARIMA: Autoregressive integrated moving average
UK: United Kingdom
ED: Emergency department
WBE: Wastewater-based epidemiology
LSTM: Long short-term memory
NYC: New York City
CNN: Convolutional neural networks
DNN: Deep neural networks
ESCC: Squamous cell carcinoma
AUC: Area under curve
IBD: Inflammatory bowel diseases
CD: Crohn’s disease
UC: Ulcerative colitis
APC: Annual percentage change
IBS: Irritable bowel syndrome
CDORPF: Comprehensive data optimization and risk prediction framework
LLMS: Large language models
DLW: Doubly labeled water
HP: Helicobacter pylori
HIPAA: Health insurance portability and accountability act
GDPR: General data protection regulation
GI-AI4H: Global initiative on AI for health
EU: European Union
LFTs: Liver function tests
PPV: Positive predictive value
